# Finishing bacterial genome assemblies with Mix

**DOI:** 10.1186/1471-2105-14-S15-S16

**Published:** 2013-10-15

**Authors:** Hayssam Soueidan, Florence Maurier, Alexis Groppi, Pascal Sirand-Pugnet, Florence Tardy, Christine Citti, Virginie Dupuy, Macha Nikolski

**Affiliations:** 1 Molecular Carcinogenesis, The Netherlands Cancer Institute, 1066CX Amsterdam, The Netherlands; 2 Univ. Bordeaux, CBiB, F-33000 Bordeaux, France; 3 Univ. Bordeaux, UMR 1332 Biologie du Fruit et Pathologie, F-33140 Villenave d'Ornon, France; 4 INRA, UMR 1332 Biologie du Fruit et Pathologie, F-33140 Villenave d'Ornon, France; 5 Anses, Laboratoire de Lyon, UMR Mycoplasmoses des Ruminants, F-69364 Lyon, France; 6 INRA, UMR1225, F-31076 Toulouse, France; 7 Univ. Toulouse, INP-ENVT, UMR1225, F-31076 Toulouse, France; 8 CIRAD, UMR CMAEE, Campus de Baillarguet, F-34398 Montpellier, France; 9 Univ. Bordeaux, CNRS / LaBRI, F33405 Talence, France

## Abstract

**Motivation:**

Among challenges that hamper reaping the benefits of genome assembly are both unfinished assemblies and the ensuing experimental costs. First, numerous software solutions for genome *de novo *assembly are available, each having its advantages and drawbacks, without clear guidelines as to how to choose among them. Second, these solutions produce draft assemblies that often require a resource intensive finishing phase.

**Methods:**

In this paper we address these two aspects by developing *Mix *, a tool that mixes two or more draft assemblies, without relying on a reference genome and having the goal to reduce contig fragmentation and thus speed-up genome finishing. The proposed algorithm builds an *extension graph *where vertices represent extremities of contigs and edges represent existing alignments between these extremities. These alignment edges are used for contig extension. The resulting output assembly corresponds to a set of paths in the extension graph that maximizes the cumulative contig length.

**Results:**

We evaluate the performance of Mix on bacterial NGS data from the GAGE-B study and apply it to newly sequenced *Mycoplasma *genomes. Resulting final assemblies demonstrate a significant improvement in the overall assembly quality. In particular, Mix is consistent by providing better overall quality results even when the choice is guided solely by standard assembly statistics, as is the case for *de novo *projects.

**Availability:**

*Mix *is implemented in Python and is available at https://github.com/cbib/MIX, novel data for our *Mycoplasma *study is available at http://services.cbib.u-bordeaux2.fr/mix/.

## Background

Moving a genome from the draft assembly stage to a complete finished genome is a labor-intensive task requiring time and further experimental work. This *finishing *step aims to improve previously assembled draft sequences that are often fragmented into hundreds of contigs. Finishing frequently requires targeted sequencing to resolve remaining issues such as misassembled regions and sequence gaps, and tries to improve coverage and accuracy in poorly covered regions of the genome. Consequently, the task of producing a complete genome requires extensive experimental work and is often out of reach for small labs. While *in silico *finishing can not resolve all of these issues, it represents a considerable speed-up of the finishing process.

Genome assembly is a lively field that has produced in the recent years numerous algorithms and tools, such as MIRA [1], CLC (http://www.clcbio.com/genomics), ABySS [2], etc. Assemblers differ in their algorithmic foundations and present different advantages and pitfalls. In addition to the sheer number of algorithmic solutions, any given assembler can be run using a number of variations of its parameter values (such as different *k*-mer sizes) and produce different results. Bring into that the fact that re-assembling an already assembled genome based on a new sequencing technology (e.g., Illumina vs Sanger) can reveal sequences that are missing in the reference assembly [3], and we end up with a very large space of easily obtainable *de novo *draft assemblies.

Armed with this observation, a number of projects aim to take advantage of either different sources of sequencing data or different assembly tools. Indeed, cross-platform data merging is advantageous because sequencing platforms have different biases [4] and thus assemblies generated from different platforms' data can complement each other [5]; [6]. Several software packages were developed in order to capitalize on different advantages of existing assemblers. Among these tools are GAM [7], minimus2 [8], MAIA [9], Reconciliator [10], Zorro [11] and GAM-NGS [12].

MAIA relies on a finished reference in order to guide the contig integration process; it is available as a MATLAB package. Zorro proceeds by masking the repeated regions known to cause problems during assembly. The minimus2 pipeline uses nucmer [13] to compute overlaps between contigs. GAM-NGS, Reconciliator and Zorro rely on reads in addition to assemblies for the merging process.

Even when there is no clearly defined reference assembly, some tools still treat the two input assemblies differently; when referring to such setup we will use the term of *asymmetry*. Graph Accordance Assembly tool GAA [14] was developed in order to improve global assembly quality starting from two assemblies, one being the target and the other being the query. It is based on the construction of an accordance graph that encodes the alignment information between the target and query assemblies. The user has to evaluate and to choose the most reliable assembly that will be the target - for which no clear-cut solution currently exists.

The specific problem that we aimed to address in current work is the high fragmentation of existing assemblies and the related to it reduced contig length. Such a problem is particularly salient for the "old" and very short (35nt to 45nt) NGS reads as for example those generated by the very first NGS chemistry of Illumina (formerly Solexa) technologies. The resulting draft assemblies are highly fragmented. The challenge in the case of *Mycoplasma *assembly project was to assemble these genomes and to develop an *in silico *finishing method in order to reduce the cost of returning back to the wet laboratory.

*Mycoplasma *are a genus of mollicutes that are a class of wall-less bacteria. Consequently, the most pertinent evaluation of assembly tools' performance is against bacterial benchmarks. A recent thorough evaluation of both assemblers and assembly merging tools has been done by Magoc and co-authors and provides a benchmark of 12 bacterial datasets called GAGE-B [15]. Most interestingly, at the end of their manuscript the authors mention that "over a large number of computational experiments, most combinations of assemblers, k-mer values, and merging algorithms did not produce improvements, and often produced inferior assemblies to the best individual assembly", with one exception where GAA produced superior results. Thus, GAA was chosen to be tested along with assembly tools in present work.

A very recent application GAM-NGS [12] attempts to merge two different assemblies by avoiding a mutual alignment step and mapping the raw reads on the two assemblies instead. The merging is based on the construction of a graph where the number of reads mapped on different regions is used to weight the edges and determine the correctness of merging two regions. An asymmetry is introduced between the two assemblies, one called the master and the other the slave, master assembly driving the merging process. Even if no clear assembly improvement is reported in the paper, since GAM-NGS was not included in the GAGE-B study, we have included it in our own benchmarking.

In the current manuscript we describe *Mix * a *finishing algorithm *that generates an assembly starting from different genome assemblies with the main objective of reducing contig fragmentation and maximizing the cumulative contig length. We address this question for the case where no reference genome is available and no asymmetry is introduced in dealing with assemblies, case not previously considered in literature. Moreover, we do not restrict ourselves to using only two assemblies. To address the excessive fragmentation, we propose a solution based on the maximal path problem in order to extend the contig length. To do this, we build a *extension graph *that connects contigs by their mutual alignments and is used to mix contigs when appropriate. We evaluate our *Mix *algorithm on GAGE-B benchmark and apply it to novel NGS data for 10 genomes from bacteria belonging to the genus *Mycoplasma*. We show that contrary to other tools that merge different assemblies, *Mix *clearly provides an advantage in terms of genome fragmentation while preserving the original assemblies characteristics in terms of missing bases and misassemblies. We argue that it is thus a good choice for *de novo *projects.

## Methods

*Mix *algorithm takes two (or more) assemblies and generates another one that *mixes *them in order to extend the length of resulting contigs. It builds an *extension graph *in which for each alignment involving extremities of two contigs we create vertices representing terminal aligned fragments. Edges of this extension graph encode how contigs are connected by an alignment. The resulting output assembly corresponds to a set of longest paths in this extension graph.

### Preliminaries

A contig *C *is composed of its identifier *id *and its sequence *s*, that is *C *= 〈*id, s*〉, its length is denoted by *|C| *and is equal to *|s|*. An assembly is a set of contigs $\text{A}=\left\{C_{i}\right\}$. Input assemblies are combined together $\text{A}=\cup {\text{A}}_{i}$, where ${\text{A}}_{i}$ are different assemblies, ideally produced by assemblers relying on different algorithmic principles.

**Definition 1 ***An *alignment *between two contigs C_i _and C_j _is a tuple a *= 〈*C_i_, C_j_, b_i_, e_i_, b_j_, e_j_, l*〉*, where b_i _and e_i _(b_j _and e_j_, respectively) are the beginning and end coordinates of the part of C_i _aligned on C_j _(of C_j _on C_i_, respectively) and l is the alignment length. A *terminal alignment *is an alignment involving extremities of C_i _and C_j_, that is at least one of the following is true*.

$$\begin{array}{c} b_{i}\in \left\{0,|C_{i}|\right\}\wedge b_{j}\in \left\{0,|C_{j}|\right\}\;\;b_{i}\in \left\{0,|C_{i}|\right\}\wedge e_{j}\in \left\{0,|C_{j}|\right\}\\ e_{i}\in \left\{0,|C_{i}|\right\}\wedge e_{j}\in \left\{0,|C_{j}|\right\}\;\;e_{i}\in \left\{0,|C_{i}|\right\}\wedge b_{j}\in \left\{0,|C_{j}|\right\} \end{array}$$

**Figure 1:**
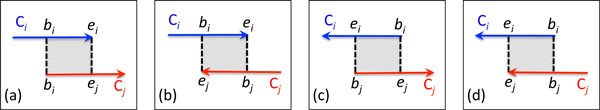
**Possible extension alignments between *C_i _*and *C_j_***. Arrows stand for contigs' orientation, *b *and *e *stand for beginning and end coordinates of the alignment on each contig. Reverse cases are not depicted (i.e. where *b *and *e *positions are inverted).

Possible terminal alignments of two contigs *C_i _*and *C_j _*are depicted in Figure 1.

An alignment set is denoted by $A=\left\{a_{i}\right\}$. The set  A is built by aligning each assembly within  A against the others. When the context does not require otherwise, we denote an alignment *a *by 〈*C_i_, C_j_*〉.

An *extension graph *is an overlap graph built over terminal alignments in  A. They are considered to have the potential to "glue" contigs, lower their number and maximize the cumulative contig length. Notice that to do this, we only consider terminal alignments, i.e. those that involve contigs' extremities. Indeed, in current work we do not question the internal logic of assembly tools that produce input contig sets.

**Figure 2:**
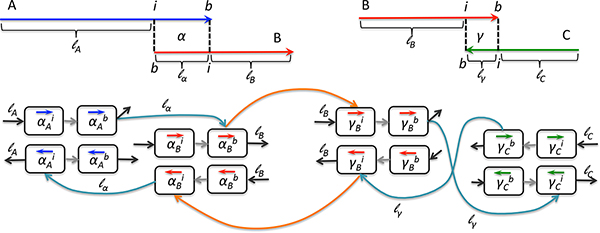
**Extension graph for two terminal alignments**. Terminal alignments *α *between contigs *A, B *and *γ *between *B, C *are each represented by eight nodes. Nodes encode the extremities of the alignment on each contig (border *b *and internal *i *extremities) and the direction in which it is read (forward *→ *or reverse *←*). Edges encode the possible "glue" between contigs. Light gray edges represent a given alignment on the contig and carry no weight. Turquoise edges connect two contigs within an alignment and are labeled by its length ( *l_α _*and *l_γ _*). Black edges connect to the In and Out nodes, allowing for reading each contig in both directions as well as complex paths and are labeled by the remaining contig length (*l_A_, l_B _*and *l_C_*). Notice that values of *l_B _*on the left-hand side of the figure and on the right-hand side are not the same as they depend on the alignment length; they are *|B| − l_α _*and *|B| − l_γ_*, respectively. Orange edges connect the extremities of different alignments in which one contig can participate: here *α *and *γ *for *B*. Their weights are deduced from the corresponding intervals (here *|B| − l_α _− l_γ _*for both).

Each terminal alignment *a *= 〈*C_i_, C_j_, b_i_, e_i_, b_j_, e_j_, l*〉 is encoded by eight vertices that correspond to the extremities of *a *on *C_i _*and *C_j_*. We distinguish boundary *b *and internal *i *locations as well as how a contig is being read (forward or reverse). Edges represent a way to "glue" *C_i _*and *C_j _*together and are weighted. Edges that connect boundary or internal nodes of different contigs carry weight equal to *l*, those that represent the remaining chunks of *C_i _*and *C_j _*carry weights equal to *|C_i_| − l *and *|C_j_| − l*, respectively. See Figure 2 for illustration. When a contig is involved in more than one alignment, its internal nodes are connected by edges, thus allowing for "gluing" more than two contigs. Weights for these edges are deduced according to the intervals defined by alignments on contigs.

To finalize the graph construction, two artificial nodes are added: In and Out. These nodes are connected to both extremities of each contig, allowing it to be read in forward and reverse direction. In this extension graph we look for paths that maximize the cumulative contig length.

### Mix algorithm

**Preprocessing **Input alignment set  A is generated by aligning each assembly in  A against all of the others. Before attempting to extend contigs, *Mix *first proceeds to clean up the alignment set  A in the following fashion.

1. If any self alignments 〈*C_i_, C_i_*〉 are present, they are eliminated.

2. If two alignments 〈*C_i_, C_j_*〉 and 〈*C_j_, C_i_*〉 covering the same region, are present in  A, only one is kept.

3. Only alignments whose length *l *is greater than a certain threshold *t_a _*are kept in  A.

4. Alignments for which *l/|s_i_| >*99% or *l/|s_j_| >*99% are eliminated from  A.

5. Alignments involving spurious contigs are eliminated; spurious contigs are those that have been aligned an abnormally high number of times. They are detected by looking for outliers in the distribution of the number of alignments per contig.

Starting with  A filtered by criteria 1-5 and  A, *Mix *proceeds to build the corresponding extension graph *G*.

**Algorithm **Once the extension graph is built we are looking for ways to traverse this graph while maximizing the path length. More formally, we identify in the extension graph the *Maximal Independent Longest Path Set *problem (MILPS) that we define as follows. Let *G *= 〈*V, E, w*, In, Out〉 be a directed weighted graph *G *with positive weights denoted by *w*(*e*) for any edge *e *∈ *E*; and let In ⊆ *V *and Out ⊆ *V *be two pre-defined sets of nodes corresponding to the entry and exit points of *G*. In general, one can add two vertices: one of in-degree 0 to serve as entry point and one of out-degree 0 to serve as exit point. A path *P *in *G *is a sequence of *|P| *vertices and we denote by *P*_(*i*) _the vertex at position *i *in *P*. A path is said to be simple if none of its vertices appears twice.

**Definition 2 ***An *Independent Longest Path Set (ILPS) *of G is a set of simple paths  P such that*

*1. *∀P∈P, *P starts in *In *and ends in *Out*, i.e. P*_(0) _∈ In *and P_|P| _*∈ Out,

*2*. ∀ *P_i_*, $P_{j}\in P$; *i *≠ *j implies P_i _∩ P_j _*⊆ In ∪ Out,

*3*. ∀ *P *of *G *from In to Out and $\forall P_{i}\in P$, either *P *and $P_{i}$ are independant, i.e. $P\cap P_{i}\subseteq \text{In}\cup \text{Out}$; or *P *is subsumed by $P_{i}$, i.e. $\sum_{k}w\left(P_{\left(k\right)}\right)\leq \sum_{k}w\left(P_{i\left(k\right)}\right)$; or *P *is not simple.

**Definition 3 ***A *Maximal Independent Longest Path Set (MILPS) *is an *ILPS  P*such that *|∪P|*is maximal over all possible ILPS, that is it covers a maximum number of vertices*.

Note that for any graph *G*, the MILPS is uniquely defined up to a relabeling of vertices. It follows immediately that if a vertex *o *∈ Out is reachable from a vertex *i *∈ In, then ∃P≠∅ and  P contains the longest simple path between *i *and *o*. Similarly, if an non empty MILPS exists for a graph *G*, it necessarily contains the longest path between In and Out.

In general, the longest path problem (LPP) is NP-hard (by reduction from a Hamiltonian path problem) and is hard to approximate. Even if a graph admits an Hamiltonian path of length *n*, it is impossible to find paths of length *n *- *n^ε ^*for any *ε >*0 unless P = NP [16]. However, trees and directed acyclic graphs are examples of non-trivial graph classes for which the longest path problem can be solved in linear time. In the same paper the authors show that LPP can be solved in polynomial time for (vertex/edge) weighted tree-like graphs. Authors of [17] proposed another practical solution for the LPP for the case of combinational circuits that contain cycles. We propose here a solution exploiting the relative tree-like structure of our extension graphs.

As we impose no restrictions on  A, we have no guarantee that the resulting extension graphs are acyclic, and thus solving MILPS is at least NP-hard. However, we empirically determined that we obtain extension graphs that are most of the time sparse and often even acyclic. Given that sparse graphs are "locally tree-like" (meaning that a typical node is not part of a short cycle) [18], a simple algorithm based on local cycle decomposition is computationally tractable. The main idea behind our algorithm is to iteratively identify longest paths in *G*, where at each iteration we work on the restriction of *G *where no elements of the previous longest path can be traversed. To compute the longest path, *G *is decomposed into acyclic and cyclic parts. The cyclic parts of *G *correspond to its strongly connected components (SCCs). For each SCC, we determine the subset of vertices that are entry and exit points; and enumerate all simple paths between them. This set of enumerated paths is then inserted in *G *in lieu of the SCC. This operation yields an acyclic graph. In such a graph, longest paths between In and Out are determined by a greedy approach based on topological ordering. The complete solution is described in Algorithm 1.

**Algorithm 1 **Maximal Independant Longest Path Set

**Require: **Directed weighted graph *G *= 〈*V, E, w*, In, Out〉

**Ensure: **Maximal Independant Longest Path Set  P

1: Let *C *be the set of non singleton strongly connected components of *G*

2: Let *R *be a mapping *V *→ *V *used to store the initial label *R*(*v*) of a vertex *v*; *R*(*V*) ← *V*

3: **if ***C *≠ ∅ **then**

4:   **for **each strongly connected component *c *∈ *C ***do**

5:      Mark entry {*v^in^*} (resp. exit {*v^out^*}) vertices of *c*, s.t. (*v, v^in^*) ∈ *E, v; * ∉*c *(resp. (*v^out^, v*) ∈ *E, υ * ∉*c*)

6:      Let *P *be the set of all simple paths from {*v^in^*} to {*v^out^*} in *c*, by performing *|c| *iterations of a breadth-first traversal rooted at the entry vertices

7:      **for **each path *p *∈ *P ***do**

8:        Insert a novel path *p*' in *G*, with $w\left(p_{\left(i\right)}^{\prime }\right)\leftarrow p_{\left(i\right)}$ and with $R\left(p_{\left(i\right)}^{\prime }\right)\leftarrow p_{\left(i\right)}$

9:      **end for**

10:   **end for**

11: **end if**

**Ensure: ***G *is acyclic

12: Let *W *be a mapping V→ℝ indicating the accumulated length *W*(*v*) down to node *v*; *W*(In) ← 0

13: Let Pred be a mapping *V *→ *V *indicating the predecessor Pred(*v*) of *v *in the current longest path; *Pred*(In) ← ∅

14: P←∅

15: **repeat**

16:    **for ***v *in the topological ordering of G starting at In and ending at Out **do**

17:      Let V′={υ′∈V|(υ′,υ)∈E, ∃ p∈P,υ′∈p∨υ′∈R(p)}

18:      Pred(*v*) *← *arg max*_v'_*_∈*V' *_(*W*(*υ*') + *w*(*v*', *v*))

19:      *W*(*υ*) *← *max*_υ'_*_∈*V' *_(*W*(*υ*') + *w*(*υ*', *υ*))

20:  **end for**

21:  Walk up the graph to construct the path *p *by letting *p ← *Pred^∗^(*Out*)

22:  $P\leftarrow P\cup \left\{p\right\}$

23: **until ***p *= ∅

24: **return **R(P), the MILPS with re-labeled vertices

In the case where *G *is acyclic, this algorithm is linear in the number of vertices. However, if *G *contains a cycle, the number of vertices that are added during the decomposition of a strongly connected component is exponential in the size of the strongly connected component. In our experiments (see Results section), we applied Mix over 300 different combinations of assemblies of bacterial genomes, and only two of them yielded assembly graphs with cycles, that were of tractable size.

Once the MILPS in the extension graph are built, we use the information stored in the graph to glue and stitch contigs corresponding to each extension path.

**Pruning **A pruning step is performed over the set of remaining contigs and extension paths. This step ensures that duplication is lowered. Indeed, one of the main drawbacks of merging two assemblies of the same genome is that most of the information is duplicated. In order to reduce this effect, we compute a coverage graph which is a directed graph with an edge between *v *and *v*' if portions of the nucleotide sequence of *v *is found in *v*'. Based on this graph, we can determine which contigs are entirely or to a large extent contained into other contigs or paths. These covered contigs are then removed from the final assembly. To build the coverage graph, we start by generating a coverage matrix that indicates how much an element of the current assembly graph (either a contig or an extension path) is covered by another element. To compute the coverage, we reuse the initial set of alignments  A to determine how many nucleotides of a source element are found in the target element. This coverage is computed for each possible pair. The coverage matrix is then thresholded to only keep pairs of elements with high coverage (90 % in our experiments). We then consider this thresholded matrix to be the adjacency matrix of a directed graph. This requires to decide the direction of inclusion for each pair where the coverage is above the threshold. This direction is determined by selecting as source the element with the highest ratio of covered nucleotides. In the case of ties, the shortest element is elected as source. This edge-orientation strategy can nonetheless produce cycles in the coverage graph as several contigs from different assemblies can be highly similar. In such cases, cycles are broken by selecting the element with the longest nucleotide sequence from each strongly connected component. We then perform a topological sort on the coverage graph and systematically remove contigs that are not covering any contig but that are covered by other contigs or paths.

## Results

### Performance evaluation

We evaluated the performance of *Mix *on the GAGE-B data set [15] against both raw assemblies and two tools that allow combining assemblies, GAA (best in GAGE-B study) and GAM-NGS (not previously evaluated). The benchmark provided by GAGE-B concerns 8 bacterial genomes. Starting point data consists of HiSeq and MiSeq Illumina reads (sometimes both, sometimes just HiSeq). Tools tested in the GAGE-B study include 8 assemblers and 2 merging algorithms, minimus2 and GAA. These latter have been chosen since they are (almost) reference free, contrary to the others. As already stated in the Background section, mixing assembly tools mostly provided inferior results, however for some cases GAA managed to improve the N50 size with an increase of *>*80%.

We applied GAA and GAM-NGS for all pairwise combinations of 8 assemblers twice (accounting for the asymmetry). We evaluated *Mix *results against those for the 8 assemblers, as well as merged assemblies produced by GAA and GAM-NGS. All evaluations were performed using QUAST [19] under the same parameters as GAGE-B. Three different types of metrics are used by QUAST. First, classical assembly statistics based on the distribution of the length of each contig of an assembly. These statistics do not require any reference genome and are used to measure the fragmentation of an assembly. A second set of statistics is derived from an alignment of the assembly against a reference genome. Contigs that are aligned over distant locations in the reference genome or that contain misassemblies are split, and fragmentation is measured over the split contigs. Using these alignments, additional measures report the ratio of duplication as well as the fraction of the reference genome that is covered by an assembly. A third, more robust, statistics is derived from the conservation of gene products. These last two statistics can be measured only if a fully assembled and annotated reference genomes is provided.

**Figure 3:**
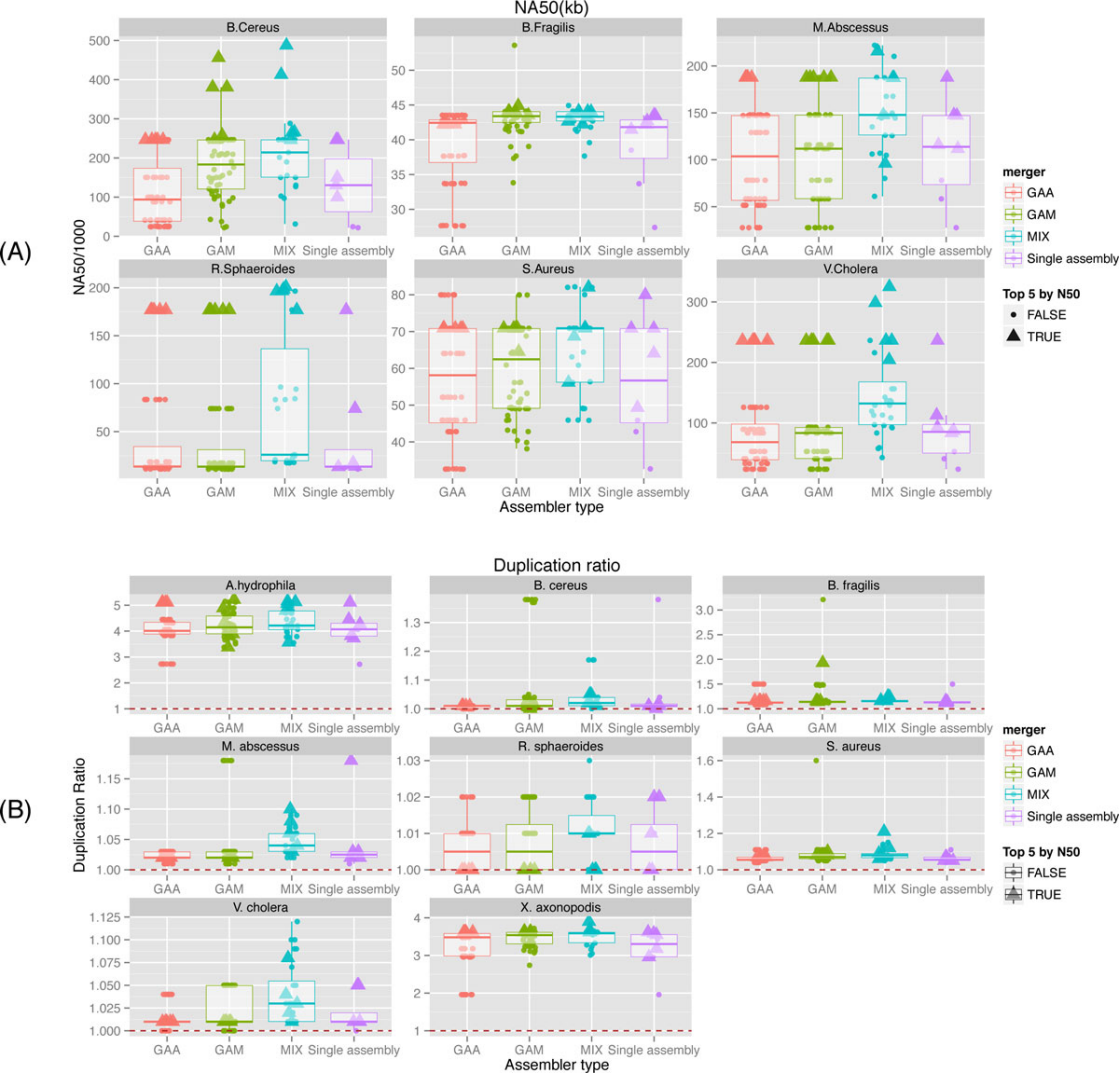
**Comparison of (A) NA50 and (B) duplication ratio measures for GAGE-B benchmark**. (A) For six bacterial genome (six panels), eight assemblies were provided by GAGE-B, and were merged either with GAA (64 combinations), GAM-NGS (64 combinations) or Mix (28 combinations only since no asymmetry between input assemblies is introduced) or not further processed (Single Assembly). The resulting assemblies were accessed against the reference genome by QUAST and the length of the shortest aligned contig from all that cover 50% of all assembly (AKA NA50 or "corrected N50") for each possible combinations of species, mergers and assembers are reported as points (Top panel). The higher the better. Box-plots indicate the quartiles of the distribution of NA50. For each species and mergers, the top 5 combinations of assemblies according to N50 were selected, and their NA50 are depicted using large triangles. Panel (B)) report the duplication ratio of the same assemblies, the horizontal dashed line indicate a perfect ratio of 1.

In total 1171 different assemblies were produced by crossing each species, master and slave assembler datasets (for GAM and GAA) and all possible pairs of assemblers for *Mix *. All original assemblies were downloaded from the GAGE-B website, they consist of contigs assemblies and correspond to HiSeq libraries, with the exception of *B. cereus *for which we used assemblies based on MiSeq libraries in order to match the GAGE-B setup. Only 13 species/merger/assemblers combination are missing from the full factorial setup. Figure 3 reports the NA50 distributions per species and assembly merger contrasted with single (unmerged) assemblies. Two species are missing from this figure, *X. axonopodis *and *A. hydrophila*, since the strain sequenced during the GAGE-B project is too distant from the reference genome to compute a NA50 value (this holds for all assemblies for these two species). In setups where a close-enough reference genome is not available, the sole statistics available to "score" assemblies are based on fragmentation measures, notably the N50. To simulate such reference-less setup, we selected for each species and each assembly merger the top 5 assemblies when ranked by N50. By analyzing how these "blindly" selected top N50 assemblies are scored with regards to statistics based on a reference genome, we can analyze the soundness of this selection heuristic.

In Figure 3, we observe that for all but *S. aureus *either GAM-NGS or *Mix *improve the single assembly substantially. Notably for *B. cereus*, for which the authors of GAGE-B already reported some improvement over single assemblies when using GAA, we manage with GAM or *Mix *to improve even more. The best *Mix *assembly for *B. cereus *stitches 90 contigs from MaSuRCA and 105 from SOAP into 47 contigs (including 4 extension paths), improving the NA50 score by 97%(NA50 of 487kb). For five out of six species, one of the top 5 assemblies generated by *Mix *is better than the best GAA, GAM and single assemblies. In particular, *Mix *significantly improves statistics measuring fragmentation of assemblies (for complete results, see results and figures available at https://github.com/cbib/MIX), as well as alignments of contigs. Similar plots and tabular data for other QUAST statistics are available on the accompanying MIX website. These also show the asymmetry in the results when one or another of assemblies is treated as target (resp., master) by GAA (resp., GAM-NGS).

Of particular concern when merging multiple assemblies is the potential increase in duplication. Indeed, the bottom panel of Figure 3 shows that overall, the mean duplication ratio for *Mix *is higher than for other assemblers, the worst case happening for *V. cholera *where one of *Mix *top 5 assemblies has a duplication that is out of range of the others. It is worth noting however that generally the duplication ratio of *Mix *assemblies stays within the same range as that produced by other assemblers (on the order of 1-2%). Finally and most importantly, we also observe that selecting assemblies solely based on the N50 value often selects the best assemblies, as validated by additional reference-genome based statistics.

### Application to Mycoplasma genomes

We have assembled the 10 newly sequenced genomes of bacteria belonging to the genus *Mycoplasma. Mycoplasmas *are small bacteria often portrayed as the best representative of the minimal cell. Indeed, their genomes are extremely reduced (*i.e*., 0.58 to 1.4 Mbp) with a low GC-content, most of them ranging from 24 to 30%. For the *Mycoplasma *genomes the available NGS data consisted in 454 and Illumina (mate paired) reads, produced in the frame of the ANR *EVOLMYCO *project (see Table 1).

**Table 1:** Summary of NGS reads volume used for genome assembly of 10 Mycoplasma genomes

Genomes	Abbreviations	454 mate pair		Illumina	
		
		#reads	med. size	#reads	size
*Mycoplasma *auris 15026	MAUR	107423	152.32	4386186	36

*Mycoplasma *bovigenitalium 51080	MBVG	132462	156.01	28752688	36

*Mycoplasma *ovipneumoniae 14811	MOVI	97641	160.49	6889585	36

*Mycoplasma *bovis 1067	MBOVb	203245	166.97	35808407	36

*Mycoplasma *mycoides subsp. capri PG3	MMC	265968	145.12	4817991	36

*Mycoplasma *capricolum subsp. capripneumoniae 99108	MCCP	150110	134.31	32510614	36

*Mycoplasma *mycoides subsp. mycoides B345/93	MSCb	247991	142.42	5342924	36

*Mycoplasma *mycoides subsp. mycoides C425/93	MSCc	186553	162.18	3585785	36

*Mycoplasma *mycoides subsp. mycoides Gemu Goffa	MSCe	132717	168.14	28776419	36

*Mycoplasma *mycoides subsp. mycoides KH3J	MSCd	163636	169.86	31781063	36


Raw data has been first processed for quality.

To build input assemblies we have chosen three assemblers: ABySS, MIRA and CLC. Two of them were chosen based on the GAGE-B study by considering the following points.

1. SPAdes [20] was the winner in terms of N50. However it produced a large number of small, unaligned contigs and was consequently excluded from our study.

2. ABySS consistently produced assemblies with the fewest errors and had the second best N50.

3. MIRA produced a large corrected N50 with errors occurring mostly in smaller contigs.

Moreover, MIRA and ABySS rely on different algorithmic methods (overlap/layout/consensus and deBruijn graph construction, respectively). Two other considerations were taken into the account for the *Mycoplasma *case-study. First, ABySS was specifically developed for very short reads, which is the case for our application (see Table 1). Second, MIRA aims at combining reads from different sequencing technologies, which is the case for the *Mycoplasma *data (Illumina and 454). We have added the CLC Assembly Cell (based on the deBruijn graph), a commercial solution that was not part of the GAGE-B evaluation, but shows high N50 statistics.

ABySS was run over a large span of k-mer values (25 to 36) for each genome and the best solution in terms assembly statistics was retained each time for further assembly combination.

**Figure 4:**
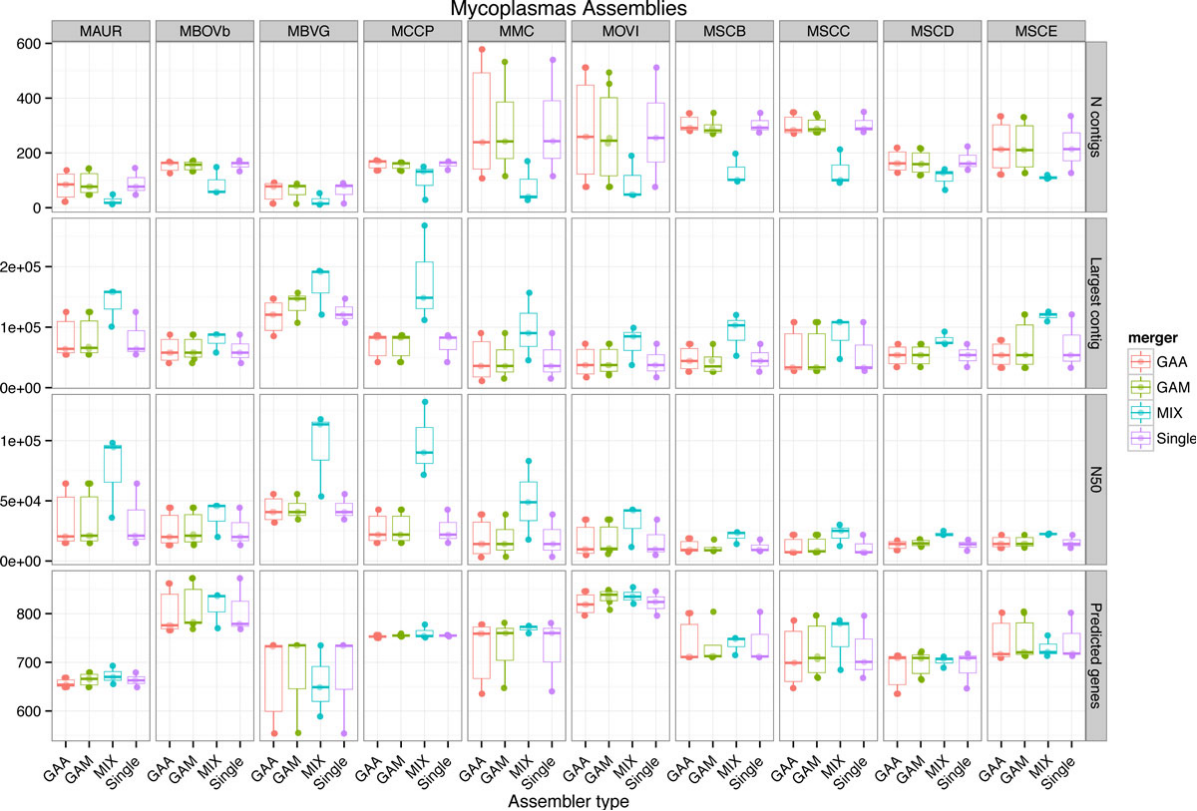
**Comparison of single and merged assemblies for *Mycoplasma***. For ten bacterial *My- coplasma *genomes (ten columns), we generated three assemblies using CLC, MIRA and ABySS, that were subsequently merged either with GAA, GAM-NGS or *Mix *(28 combinations); or not further processed (Single Assembly). The resulting assemblies were assessed using standard statistics for genome assemblies (four rows): Number of contigs, size of the largest contig, N50, number of genes of more than 300bp identified by the GeneMark gene finder. For the number of contigs, the lower the better. For the other three statistics, the higher the better.

Input assemblies for each of the 10 *Mycoplasma *genomes were combined using GAA, GAM-NGS and *Mix *. GAA and GAM-NGS were applied twice to each pair of input assemblies, owing for the asymmetry in their solutions. *Mix *was applied once to each pair of input assemblies as well as to all three input assemblies taken together. Results of these computations as well as NGS reads are available at http://services.cbib.u-bordeaux2.fr/mix/. In Figure 4 we compare these assemblies using the standard genome assembly statistics applicable when no reference genome or annotations are available. We observe that we are able to significantly reduce the fragmentation with *Mix*, as exemplified by the substantial decrease in the number of contigs as well as the size of the largest contigs. This improvement is not counterbalanced by an increased duplication or by a loss of putative functional genomic content.

**Core genome conservation **GAGE-B study was based on genomes having a complete reference sequence with known proteome. This was particularly useful in order to evaluate the biological pertinence of assembly results. Our *Mycoplasma *study is though truly *de novo *and we do not have reference genomes. However, *Mycoplasmas *are a well studied genus where a large number of genomes are fully sequenced and annotated.

This provided us the opportunity to evaluate how the core genome is preserved by assemblers and their combinations. Indeed, *core genome *is defined as the set of genes present in all strains. We have computed the core genome based on the 31 *Mycoplasma *complete genomes (Table 2) according to two criteria. First, predicted proteins from each of the genomes included in the set have to be present in each cluster. Second, one single homolog per genome has to exist to avoid paralog ambiguity. The 10 draft genomes used for our study were naturally excluded from the genome set. The computed *Mycoplasma *core genome contains 170 clusters of direct orthologs, most of which are related to basic cell machinery. The sequences of the core genome depend on the genomes already available (thanks notably to the *EVOLMYCO *project).

**Table 2:** Genomes used for the core genome computation

M. gallisepticum R High	M. gallisepticum R Low	M. gallisepticum F
M. genitalium G37	M. pneumoniae M129	M. penetrans HF-2

U. parvum 21815	U. urealyticum 33699	U. parvum 700970

M. agalactiae 5632	M. agalactiae PG2	M. bovis PG45

M. bovis Hubei	M. fermentans JER	M. synoviae 53

M. pulmonis UABCTIP	M. hyopneumoniae 232	M. hyopneumoniae J

M. hyopneumoniae 7448	M. hyorhinis HUB-1	M. mobile 163K

M. mycoides subsp. capri GM12	M. arthitidis 158L3-1	Mesoplasma florum L1

M. mycoides subsp. capri 95010	M. hominis PG21	M. leachii 99

M. mycoides subsp. mycoides PG1	M. mycoides subsp. mycoides Gladysdale	M. leachii PG50

M. capricolum subsp. capricolum 27343		


The *core genome *is defined as the set of orthologous genes present in all strains.

Since these clusters represent highly conserved sequences corresponding to essential genes, ideally, each cluster should be found in the assembled genomes. Moreover, sequence conservation should be preserved over entire length of the protein. To measure this, we first align each protein sequence of each cluster against each assembly. For each cluster, the alignment with the highest e-value is retained. Since our aim is to find entire protein/gene sequences, the alignment length is very important. Indeed, if the sequence is not entirely conserved, this can signify that it has been fragmented during assembly: either one part is located at an extremity of one contig and another part at the extremity of an another contig, or in the worse case, it can be the marker of a misassembly.

**Figure 5:**
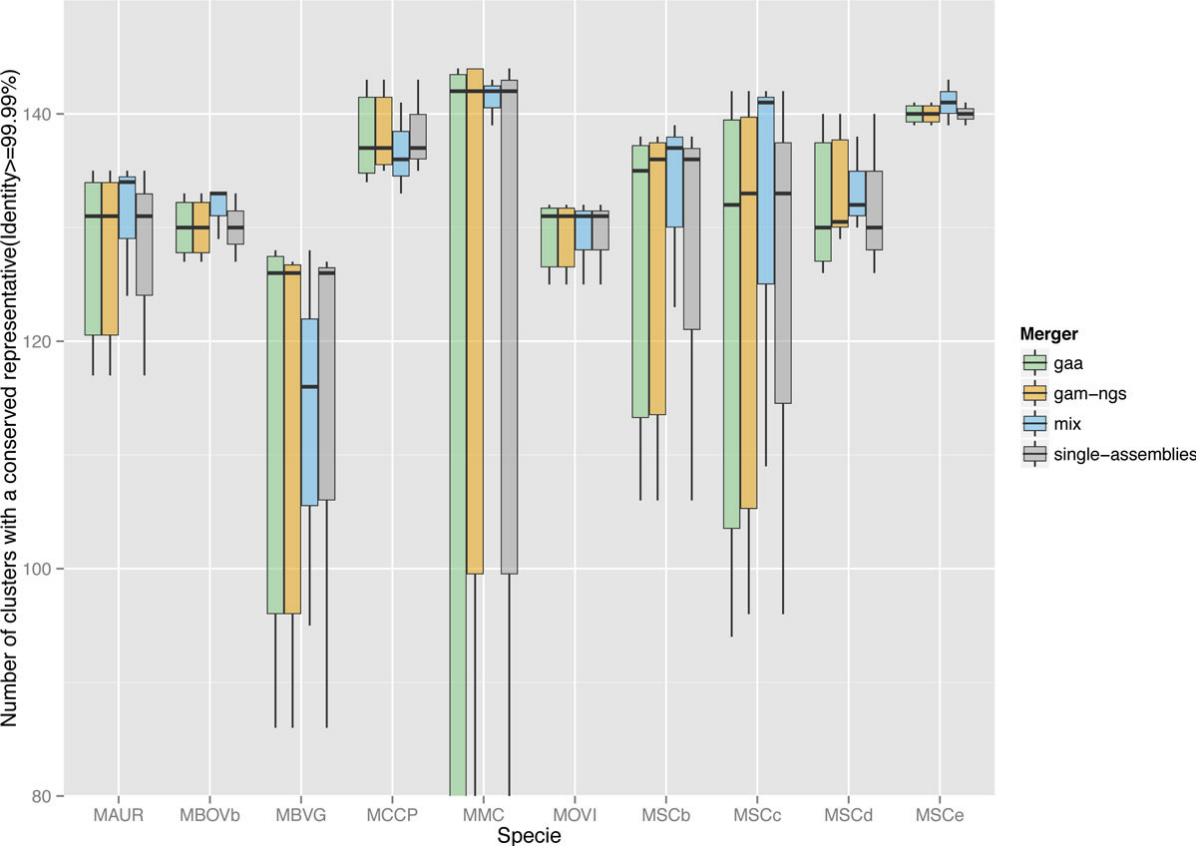
**Core genome conservation**. For ten bacterial *Mycoplasma *genomes, assembled using using CLC, MIRA and ABySS and then either left as is (single-assemblies) or combined using GAA, GAM-NGS or MIX or; we determined how much a core genome defined over the whole genus of *Mycoplasma *is preserved for these ten genomes. The core genome is a set of 170 clusters of orthologous genes present in all strains. For each combination of species, single assemblies and merger, this figure report the distribution of the number of clusters of the core genome for which we can find at least a single gene present with 99% identity in the assembly.

Hence, for each assembly and for each cluster, given the length *l_A _*of the best scoring alignment, and the length *l_P _*of the protein representing the cluster, we use the percentage of the expected length that is effectively aligned against the assembly, *l_A_/l_P_*. For a given threshold 0 *≤ t ≤ *100, we count clusters that have at least one protein that aligns with *l_A_/l_P _> t*. In Figure 5, we present the results for all the studied genomes, and for three values of *t*: 50%, 85% and 99.99%.

Notable negative cases for our approach are MCCP and MBVG, where *Mix *produced lower quality results. However, on the other cases it shows better conservation of core genome. Importantly, in the case of *Mix *this conservation is consistent between different combinations of input assemblies, as exemplified by a shorter inter-quartile range than that of other tools.

## Discussion

Despite the progress of sequencing technologies and of bioinformatics methods, *de novo *assembly of genomes remains a challenge with a lot of hurdles. The cost of sequencing falling down and the computing capacity increasing, *de novo *assemblies of genomes are released at an increasingly fast pace.

The goal of our work was to combine the strengths and to balance the weaknesses of different assembly programs in order to lower contig fragmentation. A similar goal has been previously explored by a number of papers, and in particular by the authors of GAA [14] and GAM-NGS [12]. A recent GAGE-B evaluation for bacterial genomes of assemblers and assembly mergers concluded that the latter did not provide any advantage and even sometimes worsened the results. GAM-NGS was not included in that study.

In current work we have described *Mix *, the first truly reference-free assembly merger. Our solution is based on solving the *Maximal Independent Longest Path Set *problem, known to be NP-hard, but tractable for this particular application and problem sizes. Evaluating *Mix *on the GAGE-B bacterial dataset, we show that our approach consistently lowers genome fragmentation without compromising biological relevance (ex., as measured by the alignment against the reference). Moreover, in the case of *Mix *choosing the final result based on N50 statistics maintains high assembly quality. Comparatively to *Mix*, both single assemblers as well as GAA and GAM-NGS provide poorer results in these terms. Nevertheless, a certain drawback of our approach lies in the duplication ratio. This should however be modulated by the fact that it generally stays within 1 to 2% range.

We conclude that *Mix *provides a sound approach for genome finishing in the context of *de novo *projects, where the final choice can be done based on the N50 statistics. Best resulting assemblies for *Mycoplasma *genomes are currently being annotated and will be shortly submitted to the EMBL.

## Competing interests

The authors declare that they have no competing interests.

## Authors' contributions

HS and FM have written the code and performed the evaluation on GAGE-B and *Mycoplasma *datasets. HS and MN have designed the data structure, proposed the algorithmic solution and drafted the manuscript. PSP, FT, CC and VD have constructed the core genome and validated the *Mycoplasma*'s assemblies. MN and AG have supervised the project. All of the authors have proof-read the manuscript.
